# Simultaneous monitoring of independent gene expression patterns in two types of cocultured fibroblasts with different color-emitting luciferases

**DOI:** 10.1186/1472-6750-8-40

**Published:** 2008-04-17

**Authors:** Takako Noguchi, Masaaki Ikeda, Yoshihiro Ohmiya, Yoshihiro Nakajima

**Affiliations:** 1 Cell Dynamics Research Group, Research Institute for Cell Engineering, National Institute of Advanced Industrial Science and Technology (AIST), 1-8-31 Midorigaoka, Ikeda, Osaka 563-8577, Japan; 2 Molecular Clock Project, Research Center for Genomic Medicine, Saitama Medical School, Yamane, Hidaka, Saitama 350-1241, Japan; 3 Department of Physiology, Saitama Medical School, Morohongo, Moroyama, Saitama 350-0495, Japan; 4 Division of Molecular/Cell Imaging Department of Photobiology, Hokkaido University Graduate School of Medicine, West 7, North 15, Kita-ku, Sapporo 060-8638, Japan

## Abstract

**Background:**

Luciferase assay systems enable the real-time monitoring of gene expression in living cells. We have developed a dual-color luciferase assay system in which the expression of multiple genes can be tracked simultaneously using green- and red-emitting beetle luciferases. We have applied the system to monitoring independent gene expressions in two types of cocultured fibroblasts in real time.

**Results:**

Two Rat-1 cell lines were established that stably express either green- or red-emitting luciferases under the control of the *mBmal1 *promoter, a canonical clock gene. We cocultured these cell lines, and gene expression profiles in both were monitored simultaneously. The circadian rhythms of these cell lines are independent, oscillating following their intrinsic circadian phases, even when cocultured. Furthermore, the independent rhythms were synchronized by medium change as an external stimulus.

**Conclusion:**

Using this system, we successfully monitored independent gene expression patterns in two lines of cocultured fibroblasts.

## Background

The broad application of luciferase reporter systems includes the transient assay of gene expression in cell extracts, the real-time monitoring of gene expression in living cells, and *in vivo *imaging of animals [1-6]. Luciferase reporter systems are widely used as sensitive, quantitative, and convenient measurement systems of gene expression in living cells [3]. The major advantages of luciferases over fluorescent reporters such as green fluorescent protein are that they do not require exogenous illumination because they catalyze luciferins to emit light directly, so the background emission from samples is extremely low [4,5]. The best-known luciferase is North American firefly (*Photinus pyralis*) luciferase.

Dual-color luciferase assay systems have been developed that allow the simultaneous analysis of the expression of multiple genes [7-11]. These systems use two luciferases that emit either green or red bioluminescence with a common substrate, D-luciferin. Their emissions are then separated by an optical filter [7,8,10,11]. These systems enable the simultaneous monitoring of binary components. One of the applications of these systems is to measure the expression of two genes in homogeneous cell populations, an applications that has only just started being used [11]. Another application is the measurement of single-gene expression in heterogeneous cell populations, which is expected to serve the study of the cellular communications that are important in various biological processes such as development, differentiation and cancer growth [12-14]. However, to date, there has been no dual-color luciferase assay system applied to monitoring gene expression levels in cocultured heterogeneous cells.

In mammals, the central circadian pacemaker, located in the suprachiasmatic nucleus (SCN) of the hypothalamus, coordinates physiological rhythms such as the sleep-wake cycle, the rhythms of body temperature and the release of hormones [15]. Circadian oscillations in the expression of clock genes such as *Bmal1, Per1*, *Per2*, *Cry1*, and *Cry2*, are found not only in the SCN but also in peripheral tissues and in immortalized cells [15-18]. Individual neurons in the SCN work as independent oscillators. They communicate with each other and produce a synchronized rhythm [17]. In addition to SCN neurons, individual cultured fibroblasts (Rat-1 cells) or embryonic fibroblasts have been shown to function as self-sustained oscillators using single-cell imaging of bioluminescence or fluorescence [18,19]. Whereas the rhythms of cultured Rat-1 cells undergo rapid damping, they resume after stimuli such as changing the medium and the application of dexamethasone (DEX), a glucocorticoid analog [20]. Such damping of the rhythms may be caused by a loss of synchrony among cells [18,19]. These properties of Rat-1 cells make them suitable as a model system to analyze gene expression patterns in cocultured cells.

To simultaneously study gene expression patterns in two cocultured types of cells, we have developed a real-time monitoring system of gene expression using the green- and red-emitting luciferases that we have previously reported [7]. The green-emitting luciferase was derived from a Japanese luminous beetle (*Rhagophthalmus ohbai*; λmax = 550 nm) and the red-emitting luciferase was derived from railroad worm (*Phrixothrix hirtus*; λmax = 630 nm) [7,21,22]. We established two types of stable Rat-1 cell lines that expressed either the green- or the red-emitting luciferase under the control of the *mBmal1 *promoter. Here we demonstrate monitoring of the independent circadian oscillations of these two cell lines when cocultured and confirm the hypothesis that individual Rat-1 cells act as self-sustained oscillators. The results show that this dual-color luciferase assay system serves as an effective tool to investigate independent gene expression patterns in coculture systems.

## Results

### Establishment of two stable cell lines reporting circadian rhythms using different color-emitting luciferases

To allow observation of independent circadian rhythms in cocultured cells, we chose the *mBmal1 *promoter because *mBmal1 *gene expression shows a clear circadian rhythm in cultured fibroblasts [19].

**Figure 1:**
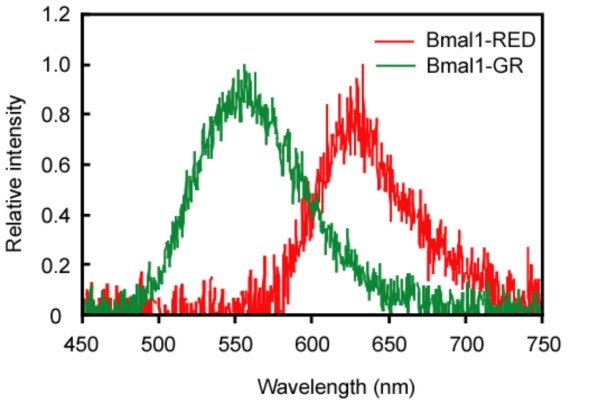
**Bioluminescence spectra of Bmal1–GR and Bmal1–RED cell lines**. Cells were incubated with 100 nM DEX for 18 h to synchronize circadian expression of the luciferases. Around the expected peak time of reporter gene expression, the Bmal1–GR (1.9 × 10^7 ^cells) and Bmal1–RED (1.7 × 10^7 ^cells) cells were trypsinized and collected from culture dishes. Cells were suspended in 250 μl of the luciferin medium. Their bioluminescence spectra were measured using an AB1850 spectrophotometer (ATTO, Tokyo, Japan). The wavelengths of the light emitted (Bmal1–GR, λmax = 556 nm; Bmal1–RED, λmax = 633 nm) were almost equal to those previously reported in cell extracts.

**Figure 2:**
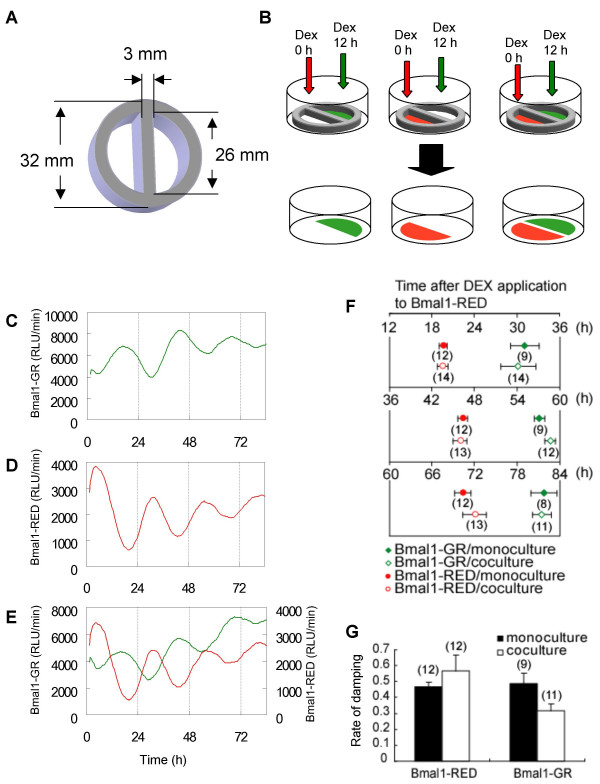
**Real-time monitoring of gene expression in cocultured Bmal1–GR and Bmal1–RED cells using the dual-color luciferase assay system**. A: Schema of the silicon frame. Silicon frames of 32 mm major diameter and 26 mm inside diameter with a 3 mm wide divider were cut from a 3 mm thick silicon plate. B: Protocol for the induction of phases separated by 12 h in Bmal1–GR and Bmal1–RED cell lines. Bmal1–GR and Bmal1–RED cells were grown in separate wells of the silicon frame. Bmal1–RED cells were treated with DEX medium (time 0 h) and Bmal1–GR cells were treated 12 h later (time 12 h). After 2 h, the medium in both wells was replaced with the luciferin medium (time 14 h). The silicon frames were removed from the dishes and the bioluminescence was measured. Representative activities of green- and red-emitting luciferases are shown for Bmal1–GR cells (C) and Bmal1–RED cells (D) in monoculture, and in coculture (E). Luciferase activities are shown on the Y axis and the measurement of time after starting bioluminescence is shown on the X axis. The independently phased circadian rhythms of luciferase activities from Bmal1–GR and Bmal1–RED were monitored even when they were cocultured in the same dish. F: Average peak times are shown as the time after the start of DEX treatment of Bmal1–RED cells. The symbols show the peak times of Bmal1–GR cells in monoculture (filled diamonds) and coculture (open diamonds); and Bmal1–RED cells in monoculture (filled circles) and in coculture (open circles). The number of samples is shown under the symbols. The bars show the SEM of the data. The phase of each cell line in coculture was identical to those in monoculture (*P *> 0.05, Mann-Whitney *U *test). G: Amplitude-damping rates of Bmal1–GR and Bmal1–RED cells in monoculture and coculture are shown. There were no significant differences in damping rates between cell lines or between monocultures and cocultures (*P *> 0.05, Mann-Whitney *U *test).

Rat-1 cell lines stably expressing either the green- or the red-emitting luciferases under the control of the *mBmal1 *promoter were generated as described in the Methods. We refer to these cell lines as Bmal1–GR and Bmal1–RED, respectively. First, we measured the bioluminescence spectra of these cells, because the spectra are the most important factors for measuring each luciferase activity by splitting the emissions. The cells were trypsinized and harvested and the spectra were measured without destroying the cells. As shown in Figure 1, the bioluminescence spectra of both cell lines are almost the same as those from cellular extracts reported in a previous study [7], demonstrating that each luciferase activity can be measured even when the cells are mixed. These cell lines showed clear circadian oscillations after treatment with DEX, which is known to synchronize circadian oscillation of clock genes in Rat-1 cells [20] (Fig. 2C, D). The circadian periods (mean ± SEM) of Bmal1–GR ± Bmal1–RED cells were 26.2 ± 0.1 h (n = 8) and 23.7 ± 0.1 h (n = 8), respectively.

### Real-time monitoring of gene expression in cocultured Bmal1-GR and Bmal1-RED cells using the dual-color luciferase assay system

If individual fibroblasts oscillate following their endogenous circadian periods, as shown previously [18,19], then the circadian rhythms of cocultured Bmal1–GR and Bmal1–RED cells would be expected to keep their endogenous rhythms. We first attempted to demonstrate that independent circadian oscillations of two types of fibroblasts could be simultaneously monitored using different color-emitting luciferases. To do this, we triggered antiphasic oscillation in the two cell lines and examined whether rhythmic *mBmal1 *expression is affected by co-culture.

Cell lines were grown separately in two wells formed by a silicon frame within a 35 mm culture dish (Fig. 2A, B). As controls, monocultures of either Bmal1–GR or Bmal1–RED cells were cultured in one of the two wells, and the other well was left empty (Fig. 2B, left and middle). The times of applying DEX to Bmal1–GR or Bmal1–RED cells were staggered by 12 h. Thus, Bmal1–RED cells were stimulated with DEX first and the Bmal1–GR cells received DEX 12 h later. The same procedure was performed on the monocultures with blank cells adjacent. During the measurement of luciferase activity, the silicon frame was removed from the 35 mm dish so that the two cell lines could establish contact through the medium. The respective luciferase activities of both cell lines were measured simultaneously. The cocultured cell lines showed antiphasic circadian rhythms of each luciferase activity and the rhythm of each cell line was identical to that of the controls (Fig. 2C–E). Reflecting the longer endogenous circadian period of the Bmal1–GR cells, the peak time of Bmal1–GR cells was delayed progressively relative to that of the Bmal1–RED cells over 3 cycles (Fig. 2F, G). There were no significant differences in peak times or rates of damping between cocultures and monocultures (*P *> 0.05, Mann-Whitney *U *test; Fig. 2F, G). Although it appears that the average rate of damping of Bmal1–GR cells was different from that of Bmal1-RED cells, the difference was not statistically significant. Thus, the varying gene expression levels in these two cocultured cell lines were monitored successfully.

### Analysis of the circadian phases of Bmal1–GR and Bmal1–RED cells cocultured with direct contact

We next examined whether signaling between neighboring cells plays a role in the synchronization of circadian rhythms. Taking advantage of the differences in circadian periods between the two types of Rat-1 cell lines, we cocultured these cells with direct contact and examined rhythmic *mBmal1 *expression.

**Figure 3:**
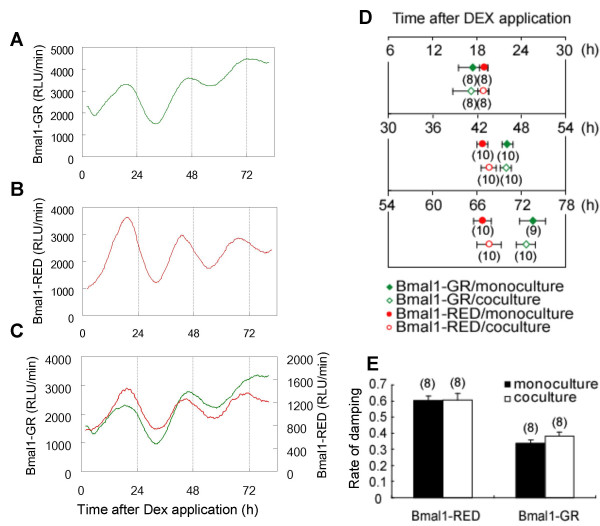
**Analysis of the circadian phases of Bmal1–GR and Bmal1–RED cells cocultured with direct contact**. The bioluminescence activities of Bmal1–GR (A), Bmal1–RED (B), and cocultures of these cells (C) were measured. The luciferase activities are shown on the Y axis and the time after starting measurement of the bioluminescence is shown on the X axis. These cultures showed clear circadian rhythms. D: Average peak times after the start of DEX treatment of Bmal1–GR cells in monoculture (filled diamonds), in coculture (open diamonds), Bmal1–RED cells in monoculture (filled circles) and in coculture (open circles). The number of samples is shown under the symbols. The bars show the SEM of the data. The phases of each cell line gradually became separated in both cocultures and monocultures. E: Amplitude-damping rates of Bmal1–GR and Bmal1–RED cells in monoculture and coculture are shown. There were no significant differences in damping rates between cell lines or between monocultures and cocultures (*P *> 0.05, Mann-Whitney *U *test).

Equal numbers of Bmal1–GR and Bmal1–RED cells were mixed and grown in a dish. As controls, monocultures of either Bmal1–GR or Bmal1–RED cells were cultured individually (Fig. 3A, B). The cells were stimulated by replacing the culture medium with medium containing DEX. Both cell lines showed clear circadian rhythms in their luciferase activities, even when cocultured with direct contact (Fig. 3C). The peak times after the start of DEX treatment and the rate of damping in amplitude were analyzed statistically (Fig. 3D, E). The peak time of Bmal1–GR cells advanced to that of Bmal1–RED cells at peak 1 but it was reversed at peak 2 and the difference was enlarged at peak 3. There were no significant differences in peak times or amplitude-damping rates between the monoculture and coculture systems (*P *> 0.05, Mann-Whitney *U *test). Thus, the independence of the circadian rhythms of Rat-1 cells was confirmed using this dual-color luciferase assay system.

### Synchronization of phases by changing the medium

To monitor the synchronization process of the fibroblasts, we changed the medium of the two cell lines oscillating in antiphase as described above, because fibroblasts are synchronized by external stimuli such as changing the medium, the application of DEX and temperature changes [18,19,23,26].

**Figure 4:**
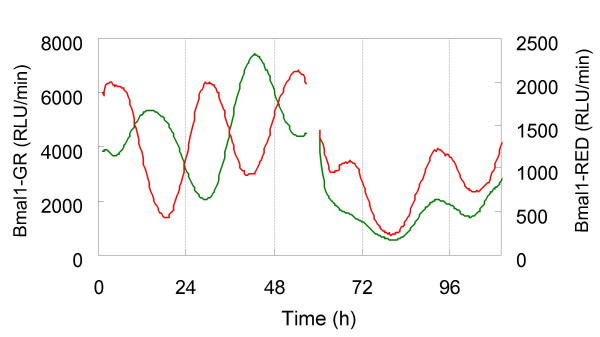
**Synchronization of the phases by changing the medium**. Antiphasic oscillations were induced in Bmal1–GR and Bmal1–RED cells, as shown in the figure. After 3 d of measurement, the medium was replaced with fresh luciferin medium. The independently phased circadian rhythms of the luciferase activities of Bmal1–GR and Bmal1–RED cells were reset by the medium change. Luciferase activities are shown on the Y axis and the time after starting measurement of the bioluminescence is shown on the X axis.

The medium was replaced 3 d after the DEX treatment of Bmal1–RED cells. After changing the medium, the independently phased circadian rhythms of Bmal1–GR and Bmal1–RED cells were shifted to a new phase and the peaks become closer to each other (Fig. 4). These results are consistent with reports that fibroblast cycles are synchronized by environmental cues rather than a coupling among fibroblasts [18,19,23]. This result shows that the phase resetting of the two cell lines could be successfully and simultaneously monitored using the dual-color luciferase assay system.

## Discussion

In this study, we report on a system for real-time monitoring of gene expression using green- and red-emitting luciferases, here used to investigate circadian rhythms in cocultured cells. There are various methods to analyze gene expression using multiple luciferases in combination, taking advantage of unique characteristics of the luciferases. Firefly and sea pansy (*Renilla*) luciferases are commonly used together to report expression levels of two different genes (e.g., the Dual-Luciferase^® ^Reporter Assay System; Promega, Madison, WI, USA). The bioluminescence of these luciferases are counted separately, using two different substrates, D-luciferin and coelenterazine, required by each luciferase. The combination of sea-firefly (*Vargula hilgendorfii*) luciferase, a secreted protein, and firefly luciferase, a nonsecreted protein, has been successfully employed to report gene expression in mouse tissues [24].

Some other dual-color luciferase assay systems have now been reported [7-10]. The green- and red-emitting luciferases of these other systems were derived from the Jamaican click beetle [9], the North American firefly [10], and the railroad worm [7,8,21]. The luciferases used in this report were derived from the Japanese luminous beetle [7,22] and the railroad worm [7,8,21]. The dual-color luciferase assay system has the following advantages: 1) the emission colors are separable by an optical filter, 2) they have a common luminescent substrate, and 3) the emission spectra are stable under physiological intracellular pH conditions [7,8,21,22]. The applications of dual-color luciferase systems have previously been limited to transient assays of gene expression in cell extracts, with the exception of one report of real-time monitoring of the expression of two genes in cyanobacteria [11]. In this report, we established real-time monitoring of gene expression in living mammalian cells. To demonstrate the further possible application of a dual-color luciferase assay system, we applied this system to monitoring gene expression patterns in two types of cocultured cell lines. In this system, furthermore, the orange-emitting luciferase can be employed as a third luciferase [7]. The third luciferase may serve to monitor the gene expression of a third cell line or to normalize the bioluminescence of the other two luciferases.

Here we demonstrated that the independent circadian oscillations of two types of cocultured stable cell lines could be reported by green- and red-emitting luciferases. These cell lines, which express green- and red-emitting luciferases under control of the *mBmal1 *promoter, showed about a 2.5 h difference in their periods. This may be caused by a positional effect arising from where the vectors are integrated in the genome rather than differences between the luciferases, as the periods of Rat-1 cells transiently transfected with these plasmids were not significantly different (data not shown). The diversity of periods among the individual fibroblasts from which these lines are derived may also cause the difference in periods [18,19]-. The separate luciferase activities of the cells in coculture were identical to those found in monocultured controls. We were also able to detect that the desynchronized circadian oscillations of stable lines became synchronized after changing the medium. In accordance with previous findings, our results confirm that these fibroblasts oscillate independently, following their individual triggered phases and intrinsic periods, and that they are transiently synchronized by external cues such as changing the medium [19,20,23]. However, we cannot rule out the possibility of coupling between fibroblasts because continuous medium replacement may be required for synchronizing factors to deliver the phase information for resetting cells.

The amplitudes of the circadian rhythms of fibroblasts cultured *en masse *undergo rapid damping, caused by a loss of synchrony [18,19]. Previous studies have investigated circadian communication between fibroblasts through mathematical modeling [18], fluorescent reporters [18] or luciferase reporters [25]. Using this dual-color luciferase assay system we could observe circadian rhythms with different phases and periods of both cell lines simultaneously. The existence of synchronizers of circadian rhythms is suggested for the SCN and other tissues because SCN and peripheral tissue explants are capable of self-sustained circadian oscillation for more than 20 cycles [26]. Coculture systems have contributed to the study of circadian communication among cells. Slices of SCN tissue induced long -lasting circadian rhythms in astrocytes [27], and immortalized SCN2.2 cells derived from the rat SCN imposed metabolic and *Per1 *gene expression in NIH3T3 cells [25]. Coculture systems using this dual-color luciferase assay system will thus help the search for circadian synchronizers in the body.

Cocultures of cells and tissues are used in many studies, for example, in studying signaling between glial cells and neurons, in examining the proliferation of cancer cells, and in observing cell differentiation [12-14]. This dual-color luciferase assay system will contribute to the analysis of the interaction of cells in many biological fields. It will add further efficiency and versatility to the study of cellular communication by enabling the simultaneous monitoring of different gene expression patterns in cells in coculture.

## Conclusion

Using this dual-color luciferase assay system, we were able to demonstrate independent circadian oscillations of *mBmal1 *expression in cocultured Rat-1 cell lines. This system is a simple and effective solution to the problems associated with conventional assays of cellular communication and can be used for any tissues or cells into which reporter gene constructs can be introduced.

## Methods

### Cell culture and generation of cell lines

F2408 (Rat-1) cells (Health Science Research Resources Bank, Osaka, Japan) were grown in Dulbecco's modified Eagle's medium (DMEM; Sigma-Aldrich, St Louis, MO, USA) with 10% fetal bovine serum (FBS; ICN Biochemicals, Aurora, OH, USA), 0.1 mg/ml streptomycin (Nacalai Tesque, Kyoto, Japan), and 100 U/ml penicillin (Nacalai Tesque) at 37°C in a humidified 5% CO_2 _incubator. Reporter plasmids, Bp/915-mGr and Bp/915-mRed, respectively expressing green- and red-emitting luciferases under the control of the *mBmal1 *promoter (-816/+99) [28], were constructed as reported [7,8]. The green- and red-emitting luciferases are commercially available as SLG and SLR (MultiReporter Assay System, Tripluc^®^, Toyobo, Osaka, Japan). For stable transfection, Rat-1 cells were cotransfected with the plasmid and pSV-Neo as the expression vector for the neomycin resistance gene using Lipofectamine 2000 (Invitrogen, Carlsbad, CA, USA) according to the manufacturer's instructions. After two days of transfection, the cells were subcultured for selection with 500 μg/ml geneticin (G418; Nacalai Tesque, Kyoto, Japan). We named the subcultured G418-resistant cell line with Bp/915-mGr as Bmal1–GR and the cell line with Bp/915-mRed as Bmal1–RED.

### Measurement of bioluminescence spectra

Bmal1–GR and Bmal1–RED were grown to confluence in 10 cm dishes. The circadian rhythms were synchronized by replacing the culture medium with DEX medium consisting of DMEM without phenol red (Gibco BRL, Gaithersburg, MD, USA) plus 10% FBS and 100 nM DEX (Nacalai Tesque). The cells were trypsinized and harvested from dishes after 18 h. Cells were centrifuged and suspended in 250 μl of luciferin medium consisting of DMEM without phenol red (Gibco), 10% FBS, 100 nM DEX, and 200 μM D-luciferin (Toyobo, Osaka, Japan). Bioluminescence spectra were measured using an AB1850 spectrophotometer (ATTO, Tokyo, Japan) without destroying the cells.

### Culture of stable cell lines

Bmal1–GR and Bmal1–RED cells were grown in DMEM supplemented with 200 μg/ml G418. To culture two types of Rat-1 cells in separate wells, silicon frames with a 32 mm major diameter and a 26 mm inside diameter with a 3 mm wide divider were cut from a 3-mm-thick silicon rubber plate (Togawa Rubber, Osaka, Japan) (Fig. 3A). The silicon frames were soaked in 70% ethanol (Nacalai Tesque, Kyoto, Japan) and placed on 35 mm dishes. After the ethanol was dried up at room temperature, the frame became stuck tightly to the bottom of a 35 mm dish. Bmal1–GR (2 × 10^5^) cells and Bmal1–RED (2.4 × 10^5^) cells were seeded in separate wells of the silicon frame. After 2–3 d, the culture medium from the Bmal1–RED cells grown to 100% confluence was replaced with DEX medium (time 0 h). Bmal1–GR cells were treated with DEX medium 12 h later (time 12 h). After 2 h of DEX treatment of the Bmal1–GR cells, the medium in both wells was replaced with luciferin medium (time 14 h). The silicon frame was removed from the 35 mm dish so that the two cell lines were in contact through the luciferin medium. The luciferin medium was covered with 2 ml mineral oil (Sigma) to prevent evaporation.

To culture Bmal1–GR and Bmal1–RED cells with direct contact, the two lines were mixed and seeded in 35 mm dishes at a density of 3 × 10^5 ^cells each. After 2–3 d, the cells at confluence were treated with DEX medium. After 2 h of DEX treatment, the medium was replaced with luciferin medium and covered by 2 ml of mineral oil.

### Real-time monitoring by dual-color luciferase assay system

The real-time monitoring of mBmal1 expression using green- and red-emitting luciferases was performed with a dish-type luminometer (AB2500 Kronos, ATTO), according to a previous report, with slight modification [7,8]. The luminometers were placed in an incubator under 5% CO_2 _in air at 20°C. The cultures were incubated at 37°C in the luminometers and bioluminescences were monitored for 1 min at 10–20 min intervals in the absence or presence of a 620 nm long-pass filter (R62 filter, Hoya, Tokyo, Japan) [7,8]. The bioluminescence count obtained was expressed in relative light units (RLU). Each green- and red-emitting luciferase activity was calculated from the total RLU (F0) and the RLU passed through the R62 filter (F1), as reported [7]. Briefly, the light intensity of green-emitting luciferase (G) and red-emitting luciferase (R) were obtained from F0 and F1 and from the optical filter's transmission coefficients for the green- (κ_G_) and the red-emitting (κ_R_) luciferases using the following simultaneous equation.

$$\left(\begin{array}{c} F0\\ F1 \end{array}\right)=\left(\begin{array}{cc} 1.0 & 1.0\\ \kappa_{G} & \kappa_{R} \end{array}\right)\left(\begin{array}{c} G\\ R \end{array}\right)$$

### Analysis of circadian rhythms in bioluminescence

To determine the periods, we analyzed the data over 4 d by subtracting a 24 h moving average from the crude data and obtained a best-fit cosine curve for the data using a least squares spectrum method [29]. To determine the peak times and amplitude-damping rate, serial records of bioluminescence were smoothed using a 10-point (2–3 h) moving average [15]. The circadian peaks and troughs were calculated as the highest and lowest points of the smoothed data. We determined peaks 1, 2, and 3 as the highest points that appeared 10.0–25.0 h, 40.0–55.0 h, and 60.0–80.0 h after the start of DEX treatment, respectively, and troughs 1, 2, and 3 as the lowest points following peaks 1, 2, and 3, respectively. The RLU difference between peak 1 and trough 1, and peak 2 and trough 2 were determined as amplitude 1 and amplitude 2, respectively. The ratio of amplitude 2 to amplitude 1 was determined as the amplitude-damping rate.

## Authors' contributions

TN carried out the experiments on measuring bioluminescence, designed experiments, analyzed data, and drafted the manuscript. MI cloned *mBmal1 *and its critical promoter region. YO initiated the project and helped in writing the manuscript. YN constructed cell lines, established the dual-color luciferase assay system, and supervised the experiments. All authors read and approved the final manuscript.
